# Understanding vegetation changes in northern China and Mongolia with change vector analysis

**DOI:** 10.1186/s40064-016-3448-y

**Published:** 2016-10-12

**Authors:** Xiaohe Gu, Weiguo Li, Lei Wang

**Affiliations:** 1 Beijing Research Center for Information Technology in Agriculture, Beijing, 100097 China; 2 Jiangsu Academy of Agricultural Sciences, Nanjing, 210014 China

**Keywords:** Change vector analysis (CVA), NDVI time series, Vegetation dynamic, CV magnitude

## Abstract

In recent years, a close link between vegetation change and climate change has been established. Vegetation change can be detected with remotely sensed images, especially with normalized difference vegetation index time series records. We used change vector analysis, especially change vector magnitude (CV magnitude), as an indicator to better understand vegetation change. Twenty-one layers of CV magnitude for each 10-day period from April to October have been acquired. Maxima, range, standard deviation, mean, and minima of CV magnitude were obtained and analyzed, identifying 11 regions with different types of vegetation change during different 10-day periods. In addition, the months of maximum CV magnitude were determined to help predict future vegetation change. The following conclusions were drawn: (a) CV magnitude can serve as an indicator to compare vegetation conditions among different years; (b) 11 typical regions were identified in the study area that show vegetation changes between 1999 and 2006; (c) the months with maximum CV magnitude can be used to better understand the key periods of vegetation change during the growing season from April to October.

## Background

Vegetation change describes temporal and spatial variations of vegetation growth. In recent years, the study of vegetation change has been suggested as one of the major ways to track global climate change. Thus, the importance of mapping and monitoring characteristics of vegetation change has been recognized as a key element in climate change studies at global and regional scales.

In the past, studies of vegetation change were based on traditional methods such as field observations, which made it possible to assess point or local vegetation change with high accuracy, but were limited in understanding large-scale changes. With the development of earth observation and satellite technology, remote sensing has provided an efficient source of data, especially for vegetation index data from which vegetation change information can be extracted efficiently and cheaply for large areas (Dengsheng 2006; Bao et al. 2014; Zhou et al. 2015). The normalized difference vegetation index (NDVI), which is derived from reflectance measurements in the red and infrared portions of the electromagnetic spectrum, is a vegetation index that shows leave volume, utilizing characteristic reflection and absorption of chlorophyll in plants (Pinty and Verstraete 1992; Leprieur et al. 2000). Higher NDVI values indicate higher concentrations of green vegetation, while lower NDVI values indicate less vegetated areas. Phenological variations of vegetation or ecosystem changes will have an impact on vegetation coverage, which is reflected in a change of the NDVI value. Animations using NDVI time series data can show the seasonal change of vegetation. As one of the important vegetation dynamic detection programs, the SPOT-VEGETATION program allows daily monitoring of terrestrial vegetation cover at regional or global scales (Trichon et al. 1999). The instrument and associated ground services for data archival, processing, and distribution have been operational since April 1998. Processing of SPOT-VEGETATION data includes improved navigation, atmospheric correction, reduced geometric distortions, and improved radiometric sensitivity (Gobron et al. 2000; Rasmus et al. 2006). SPOT-VEGETATION data have been widely used for detecting vegetation change, land use/cover, and vegetation conditions. Stephen et al. (Boles et al. 2004) conducted a land cover characterization of temperate East Asia using SPOT-VEGETATION data. Immerzeel et al. (2005) analyzed the interaction between precipitation and land use in Tibet using SPOT-VEGETATION S10 NDVI time series and Vebesselt et al. (2007) monitored herbaceous fuel moisture content with SPOT-VEGETATION time series for fire risk prediction in savanna ecosystems.

*Change vector analysis* (CVA) is a useful method for detecting vegetation cover changes, because it not only avoids shortcomings of approaches based on classification methods such as accumulated errors in individual data classifications, but can also detect more changed pixels in all bands, and can provide “from–to” change category information, which can be employed for understanding vegetation change through multi-temporal vegetation index data (Lambin and Strahler 1994). Although the change vector (CV) analysis is not a new method in understanding the vegetation dynamics, it could give rapid way in tracking the vegetation dynamic, and the ways in using the CV to obtain certain vegetation phenomena was therefore still useful to carry out vegetation dynamic (Ye et al. 2016). Jin et al. (2001) employed CVA to understand land use/cover changes using double windows flexible pace searching for CV magnitude threshold determination; Bayarjargal et al. (2006) derived drought indices using CVA with NOAA advanced very high resolution radiometer (NOAA-AVHRR) data.

**Fig. 1:**
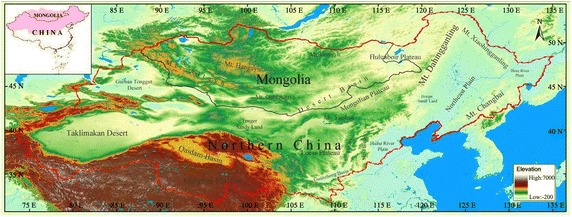
Location of the study area

In this study, SPOT-VEGETATION NDVI time series data for 1999–2006 were chosen for understanding vegetation change. In the following sections, we first define a decadal temporal vector, then calculate the magnitude of the decadal CV for each pixel in study area to derive the decade during which the greatest changes occurred. Finally, we use these results to understand vegetation change in the study area (Fig. 1).

## Study area

The study area, which consists of northern China and Mongolia, is located between 73°–136°E and 31°–54°N, spanning the transition region from semi-humid continental monsoon climate to typical dry continental climate. The region features obvious regional differences in climate and vegetation types. There are large areas of desert, desert steppe, steppe, forest steppe, and forest. Northeast Asian sandstorms originate from five regions within the study area, including sandy areas in central Inner Mongolia and adjacent regions of Mongolia, the Gobi Desert in the Xinjiang and Gansu provinces, western Inner Mongolia and adjacent southwestern areas of Mongolia, the Gobi Desert in southern Mongolia, including adjoining areas of northern Inner Mongolia, deserts around the middle reaches of the Yellow River, and the area surrounding the Taklimakan Desert (Han et al. 2010, 2014). These conditions make the study area ideal for understanding vegetation changes.

## Data and data processing

**Fig. 2:**
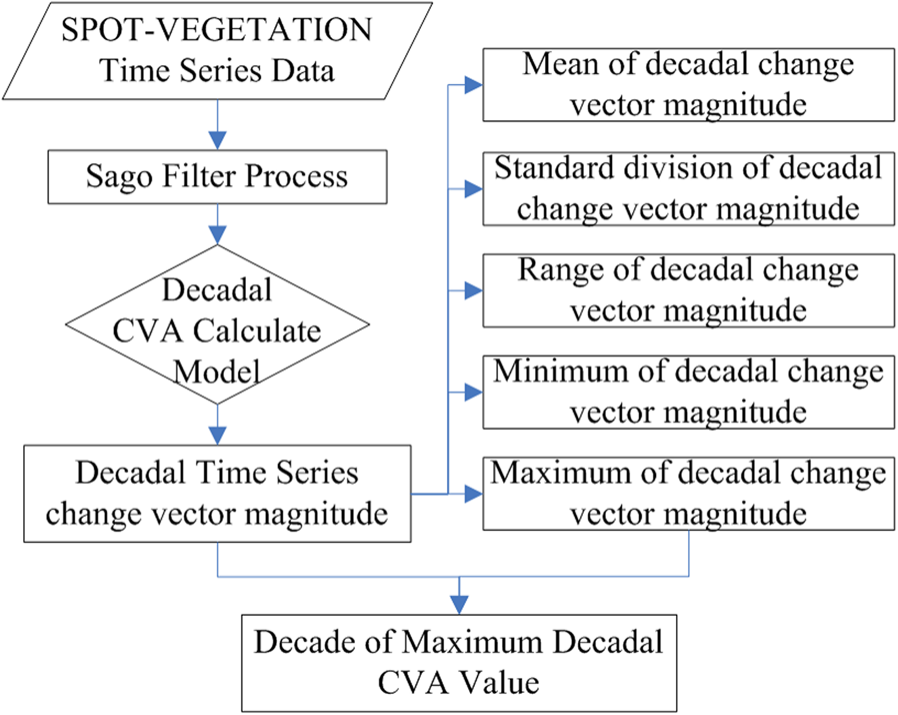
Flowchart illustrating the data processing steps

As mentioned above, the VEGETATION program allows daily monitoring of terrestrial vegetation cover through remote sensing at regional to global scales. The first VEGETATION instrument has been part of the SPOT 4 satellite since 1998 and a second instrument, VEGETATION 2, is now operational onboard SPOT 5. In this study, 8 years of SPOT-VEGETATION S10 data, from 1999 to 2006, have been processed as illustrated in Fig. 2.

CV analysis is based on multi-band images of high spatial resolution. Here, we defined a temporal vector in order to analyze time series NDVI data with high temporal resolution.

### Ten-day period temporal vector

In a first step, the same 10-day period in every year was used, with eight successive NDVI images available from 1999 to 2006 for each 10-day period. Second, the value used by the indicator under consideration ban be represented by a point in an eight-dimensional temporal space and is defined by a vector. Therefore, the same 10-day period in every year from 1999 to 2006 can be written for each pixel as an eight-dimensional temporal vector:

1 $$Q(j,y) = \left[ {\begin{array}{*{20}c} {y(d_{1} )} \\ {y(d_{2} )} \\ \cdots \\ {y(d_{8} )} \\ \end{array} } \right]$$

where $Q(j,y)$ is the multi-temporal vector for the pixel *j* in the 10-day period *y*, *y*(*d*) are the values of the indicator under consideration for pixel *j* for the 10-day period in 1999 to the same period in 2006.

### Ten-day periods change vector

Based on the definition of 10-day periods vector, any NDVI change between the same 10-day periods in different years can be described by a CV in an eight-dimensional space using the following equation:

2 $$\Delta Q(i) = \left[ {\begin{array}{*{20}c} {y_{2} - y_{1} } \\ {y_{2} - y_{1} } \\ \cdots \\ {y_{8} - y_{7} } \\ \end{array} } \right]$$

where Δ*Q*(*i*), the CV for pixel *j* between two 10-day periods, contains all changes of pixel *j* in every temporal dimension. The magnitude of the CV, $\left\| {\Delta P} \right\|,$ calculated by the Euclidean distance between the two vectors, measures the intensity of the change in NDVI.

3 $$\left\| {\Delta Q} \right\| = \sqrt {(y_{2} - y_{1} )^{2} + (y_{3} - y_{2} )^{2} + \cdots + (y_{8} - y_{7} )^{2} }$$

### Month of maximum CV magnitude

Based on the time series results of the 10-day CV magnitude, the 10-day period of maximum CV magnitude in the study area is calculated, and, combined with the 10-day periods of each month, which results in the month of maximum CV magnitude.

4 $$D_{\mathrm{max} } = Max\left( {D_{\Delta Q(i)} } \right)$$

5 $$M_{\mathrm{max} } = \left\{ {\begin{array}{*{20}l} 4 \hfill & {when{:}\,D_{\hbox{max} } = 10\,or\,11\,or\,12} \hfill \\ 5 \hfill & {when{:}\,D_{\hbox{max} } = 13\,or\,14\,or\,15} \hfill \\ 6 \hfill & {when{:}\,D_{\hbox{max} } = 16\,or\,17\,or\,18} \hfill \\ 7 \hfill & {when{:}\,D_{\hbox{max} } = 19\,or\,20\,or\,21} \hfill \\ 8 \hfill & {when{:}\,D_{\hbox{max} } = 22\,or\,23\,or\,24} \hfill \\ 9 \hfill & {when{:}\,D_{\hbox{max} } = 25\,or\,26\,or\,27} \hfill \\ {10} \hfill & {when{:}\,D_{\hbox{max} } = 28\,or\,29\,or\,30} \hfill \\ \end{array} } \right.$$

where *D*_max_ is the 10-day period when the maximum CV magnitude occurred, $D_{\Delta Q(I)}$ is the date when $\Delta Q(i)$ occurred, and *M*_max_ is the month of maximum CV magnitude.

## Results and discussion

**Fig. 3:**
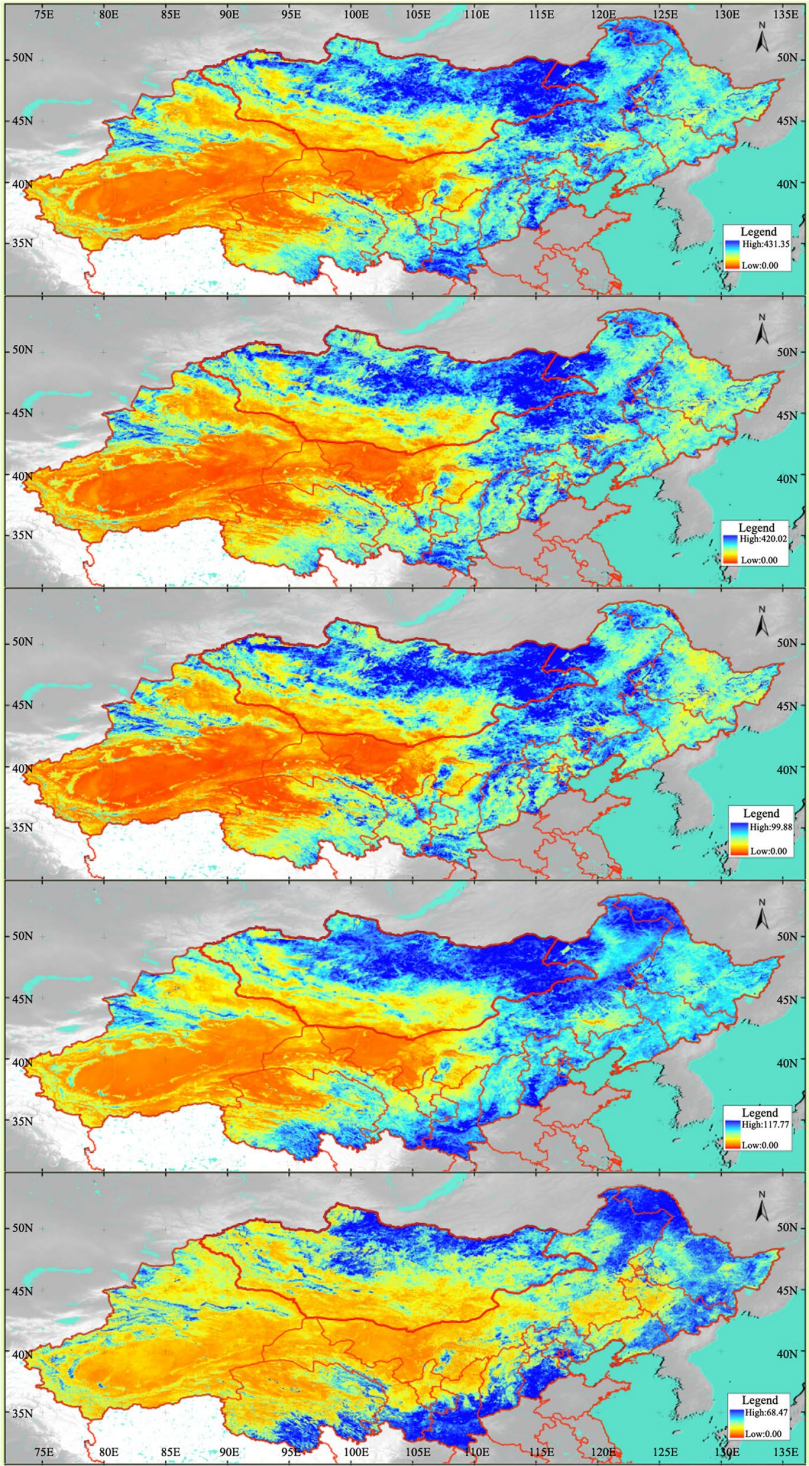
Results of the CV magnitude analysis. From *top* to *bottom* maximum CV magnitude; range of CV magnitude; standard deviation of CV magnitude; mean of CV magnitude; minimum of CV magnitude

After the CV magnitude for the growing season (April–October) is determined, several indices, including the maximum, range, standard deviation, mean, and the minimum of the CV magnitude, as well as a typical curve of certain regions, can be obtained to better understand vegetation changes in the study region (Fig. 3).

### CV magnitude analysis for understanding vegetation changes in typical regions

The maximum, range, and standard deviation of CV magnitude are higher in the east than in the west, and higher in the north than in the south of the study area. The region from Mt. Hangayn and Mt. Henteyn to the Hulunbuir Plateau and Mongolia Plateau, the area north and east of Mt. Dahingganling, Mt. Yinshan, Mt. Luliang, Mt. Tianshan, the Guanzhong Basin, and to the west of the Haihe River plain have much higher values than the other regions. The region from the Taklimakan Desert to the Qaidam Basin, from the Gurban Tonggut Desert to the Tenger Desert, and the area north of the Loess Plateau have lower values than the other regions.

Mean CV magnitudes are higher in the east than in the west, and higher in the north than in the south of the study area. The region from Mt. Hangayn and Mt. Henteyn to the Hulunbuir Plateau and Mongolia Plateau, Mt. Dahingganling, the south of Gansu Province, and the areas west of the Haihe River plain and Guanzhong Basin have higher values than the other regions. The region from the Taklimakan Desert to the Qaidam Basin, from the Gurban Tonggut Desert to the Tenger Desert, and the northern part of the Loess Plateau have lower values than the other regions.

Minimum CV magnitudes are higher in the north, south, and northeast than in the other regions. Minima in the region from Mt. Hangayn to Mt. Henteyn, Mt. Dahingganling, Mt. Xiaohingganling, Mt. Changbai, the Haihe River plain, Guanzhong Basin and the southern part of Qinghai Province are much higher than in the other regions.

There are several different regions that show vegetation change characteristics in the study area. Table 1 lists 24 different regions that were selected for investigating 11 different types of vegetation change based on their CV magnitude characters.

**Table 1:** CV magnitude characters in different regions

Region	Value						
	Maximum	Range	Standard division	Minimum	Mean	Type	Vegetation condition
Mt. Changbai	Medium	Medium	Low	High	Medium	A	D, E
Mt. Xiaohingganling	Medium	Medium	Low	High	Medium	A	C, D, E
Mt. Dahingganling	Medium	Medium	Medium	High	High	B	C, D, E
Northeast Plain	Medium	Medium	Medium	Low	Medium	C	A, B
Mt. Altayn	Medium	Medium	Medium	Low	Medium	C	G
Western Loess Plateau	Medium	Medium	Medium	Low	Medium	C	F, I
Horqin Sandy Land	Low	Low	Low	Low	Low	D	E
Desert Basin	Low	Low	Low	Low	Low	D	I
Eastern Loess Plateau	Low	Low	Low	Low	Low	D	F, I
Qaidam Basin	Low	Low	Low	Low	Low	D	I
Taklimakan Desert	Low	Low	Low	Low	Low	D	H
Tenger Sandy Land	Low	Low	Low	Low	Low	D	H
Gurban Tonggut Desert	Low	Low	Low	Low	Low	D	H
Mt. Gobi Altayn	Low	Low	Low	Low	Low	D	G
Hulunbuir Plateau	High	High	High	Medium	High	E	F
Mt. Henteyn	High	High	High	High	High	E	C, D
Haihe River Plain	High	High	High	High	High	E	B
Guanzhong Basin	High	High	High	High	High	E	A, B
Mt. Hangayn	Medium	High	Medium	High	High	F	C
Mt. Tianshan	High	High	High	Medium	Medium	G	G
Mongolia Plateau	High	High	High	Low	Medium	H	F
Mt. Yinshan	High	High	High	Low	Medium	H	F
Mt. Taihang	Medium	Medium	Low	Medium	Medium	I	E
Mt. Luliang	Medium	Medium	High	Low	Medium	J	E
Qinghai Plateau	Medium	Low	Medium	Medium	Medium	K	F

High: high magnitude value, medium: medium magnitude value, low: low magnitude value, A: year ripe grain crops and cold resistant industrial crop, B: 1 year two ripe grain crops or 2 years three ripe grain crops, C: evergreen needleleaf forest, D: deciduous broadleaf forest, E: closed shrubland, F: prairie and sparse tree bush prairie, G: meadow and herbaceous bog, H: desert, I: non-vegetation

#### Type A

**Fig. 4:**
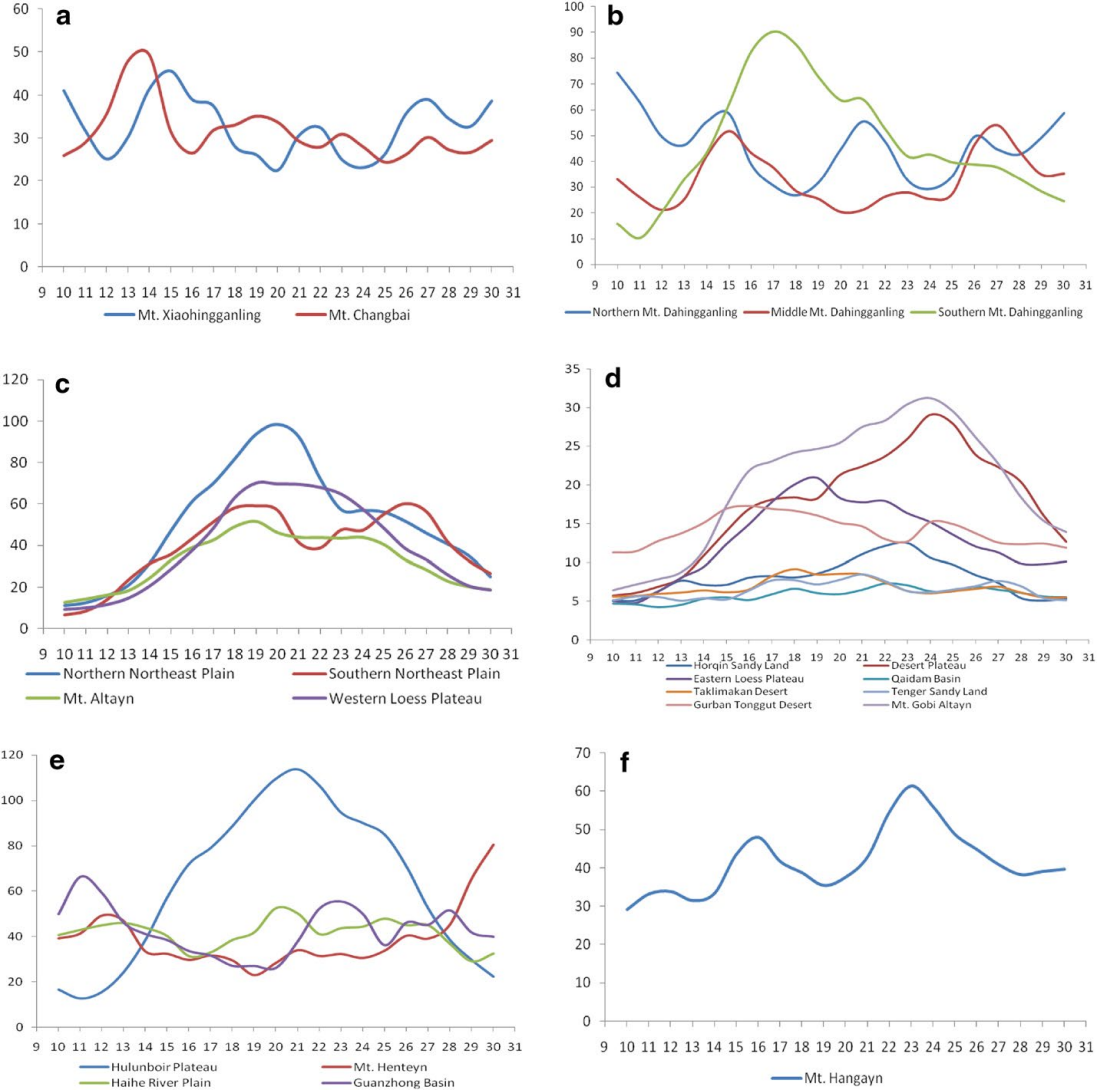
CV magnitude time series for different regions. All the X-axis are the 10-day’s order in a year, while all the Y-axis are the CV magnitudes. **a** Type A, **b** Type B, **c** Type C, **d** Type D, **e** Type E, **f** Type F in Table 1

The maximum, range, and mean show medium magnitudes, while the minimum shows high magnitudes, and the standard deviation shows low magnitudes. There are two regions of this type, Mt. Changbai and Mt. Xiaohingganling, which are mostly covered by evergreen needleleaf forest, deciduous broadleaf forest and closed shrubland that show little change during the same 10-day periods of different years during the growing (April–October). Steady vegetation growth during the growing season causes this vegetation change character. Figure 4a shows that the two regions reach their maximum CV values in April and May and that the CV values remain between 20 and 40 during the summer and autumn periods, which indicates that these regions do not undergo large changes in every 10-day period during the growing season. In addition, Mt. Xiaohingganling’s maximum CV values occur later than those of Mt. Changbai, which means that this method can also detect vegetation phenology differences.

#### Type B

The maximum, range, and standard deviation are at a medium level, while the minimum and mean CV values are high. Only Mt. Dahingganling belongs to this type. This region’s vegetation is characterized by deciduous broadleaf forest and closed shrubland in the southern part and by evergreen needleleaf forest and deciduous broadleaf forest in the northern part. To further investigate this region, three subregions were highlighted. In the south of Mt. Dahingganling, CV magnitude changes distinctively in June and the CV curve is different from the north and middle Mt. Dahingganling region, which may be because the vegetation in the southern part is closed shrubland that differs from the other parts of the region. In the south and middle part, the trend of CV magnitudes is similar to Type A, which may be because those regions have almost the same vegetation, with maximum changes occurring in May or July.

#### Type C

This type is characterized by low CV minima but all other CV characters are at a medium level. The northern Northeast Plain, Mt. Altayn, and the western Loess Plateau belong to this type. This region is covered by farmland, meadow and herbaceous bog, as well as prairie and sparse tree bush prairie. The CV minimum is lowest during the growing season, when there is no vegetation. Four regions belong to this type (Fig. 4c). The northern Northeast Plain shows one peak compared with the southern Northeast Plain, which has two peaks due to different types of farmland. The north is characterized by annual crops, while 2 year three ripe crops or 1 year two ripe crops dominate in the south. Mt. Altayn and the western part of of Loess Plateau show almost the same vegetation change trend, although the maximum CV values for the western part of the Loess Plateau are higher than those for Mt. Altayn, which indicates that vegetation on the Loess Plateau changes more recognizable than that of Mt. Altayn.

#### Type D

All CV magnitudes are low. There are nine regions that belong to this type: the Horqin Sandy Land, the Desert Basin, the Loess Plateau, the Qaidam Basin, the Taklimakan Desert, the Tenger Desert, the urban Tonggut Desert, and Mt. Gobi Altayn. All those regions are covered by desert or sandy land, which have almost no vegetation for the entire year, resulting in CV magnitudes of <30 or even <10 in some regions. Within this type, eight regions were selected to better understand the relationship between vegetation change and CV magnitude. Mt. Gobi Altayn and the Desert Plateau are characterized by similar change types during the growing period, with maximum CV magnitudes in August or September. The eastern Loess Plateau falls into a second group, with changes similar to the western Loess Plateau, but lower CV magnitudes. The third group consists of the Gurban Tonggut Desert, Horqing Sandy Land, Qaidam Basin, Taklimakan Desert, and Tenger Sandy LandDesert. Most of these regions are vegetation-free, which is the reason why CV magnitudes are lower than in the other region and the curves are smooth.

#### Type E

All CV magnitudes are high, only the minimum for Hulunbuir Plateau is medium. Four regions, the Hulunbuir Plateau, Mt. Henteyn, the Haihe River plain, and the Guanzhong Basin, belong to this type. The Hulunbuir Plateau is covered by prairie and sparse tree bush prairie, resulting in a relatively large portion of the area covered by vegetation, but still less than areas covered by evergreen needleleaf forest and deciduous broadleaf forest, causing lower minima. For Mt. Henteyn, which is covered by evergreen needleleaf forest and deciduous broadleaf forest, all magnitudes are high. The Haihe River plain and Guanzhong Basin, which are covered by annual crops, 2 year three grain crops and 1 year two grain crops, are characterized by continuously vegetated surfaces from April to October and are affected by human activity, which causes CV magnitudes that are higher than in other regions. The CV magnitude time series curve for this type (Fig. 4e) shows that vegetation on the Hulunbuir Plateau changes more significantly during July and August, while the other regions remain relatively steady during the summer but change somewhat more in early spring and late autumn.

#### Type F

Maximum and standard deviation CV values are at a medium level, while range, minimum, and mean show high values. Only Mt. Hangayn belongs to this type. Although this region is covered by evergreen needleleaf forest, its CV magnitude curve (Fig. 4f) shows a first peak in May or June and a second peak in August, which means that these two periods are important for understanding vegetation change in this region.

#### Type G

**Fig. 5:**
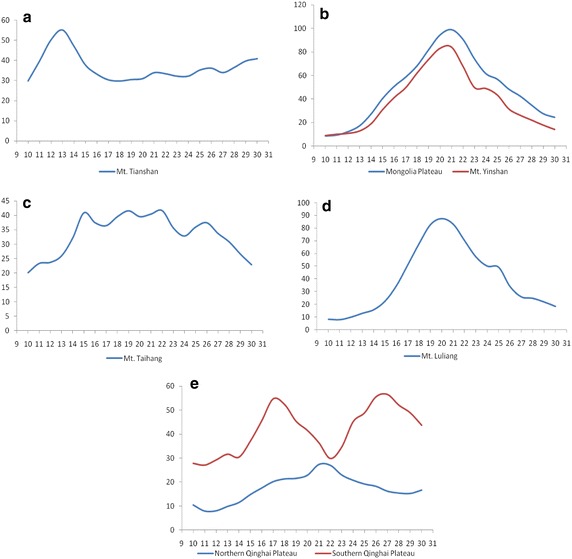
CV magnitude time series for different regions. All the X-axis are the 10-day’s order in a year, while all the Y-axis are the CV magnitudes. **a** Type G, **b** Type H, **c** Type I, **d** Type J, **e** Type K in Table 1

CV maxima, range, and standard deviation are high, while minima and mean are at a medium level. Only Mt. Tianshan belongs to this type. From its CV magnitude time series curve (Fig. 5a), we can conclude that vegetation in this region changes predominately during spring, which is the region’s main growing season.

#### Type H

Maximum, range, and standard deviation are high, while the minimum is low level, and the mean is at a medium level. Two regions, the Mongolia Plateau and Mt. Yinshan, belong to this type. From Fig. 5b we can conclude that vegetation change for this type is greatest during July and August, which makes these months the key vegetation change period for those regions.

#### Type I

Maximum, range, minimum, and mean are at a medium level, while the standard deviation is low. Only Mt. Taihang belongs to this type. From its CV magnitude time series curve (Fig. 5c), we can conclude that this region remains steady from June to September and has lower CV values in April, May, and October. From June to September, the CV magnitude remains above 35, which means that vegetation change is greater during those months, making this period the key period for vegetation change.

#### Type J

CV values for maximum, range, minimum, and mean are at a medium level, while the standard deviation is high, and the minimum is low. Only Mt. Luliang belongs to this type. Based on the CV magnitude curve (Fig. 5d), vegetation change reaches its peak in the summer, especially in June, making this month the key period for vegetation change during the region’s growing season.

#### Type K

Maximum, standard deviation, minimum, and mean are at a medium level, while the range is low. Only the Qinghai Plateau belongs to this type. On the Qinghai Plateau, two regions were selected for analyzing vegetation change. One is the southern part of the Qinghai Plateau, where the CV time series curve shows two peaks, one in June and one in September, making those 2 months important for vegetation change in this area. The other region is the northern part of the Qinghai Plateau, which only shows one peak in July. The CV magnitude for this area remains below 30 during the entire growing season, because this area does not have much vegetation cover.

The following conclusions can be drawn from the above analysis. First, CV magnitude can serve as an indicator to detect vegetation in different regions with different vegetation conditions and different phenologies. Second, in some regions this method can help understand vegetation phenology and identify key periods of vegetation change during the growing season.

**Fig. 6:**
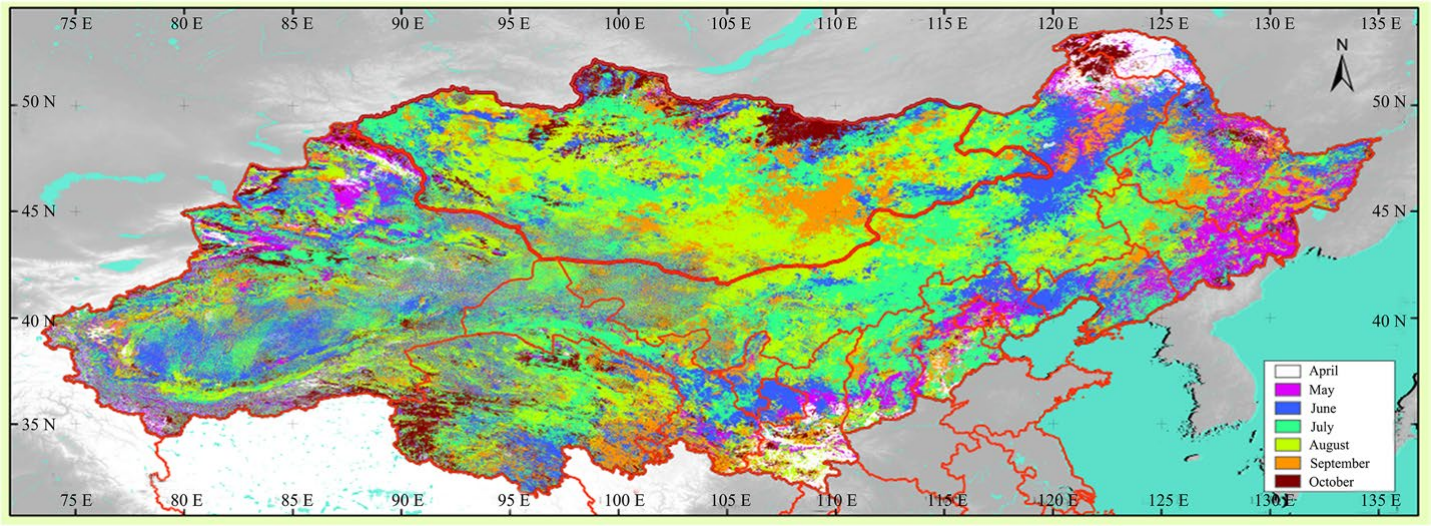
Months of maximum CV magnitude

Based on our analysis, the month of maximum CV magnitude appears most important for understanding vegetation type and its phenology over the course of a year, which may facilitate decision-making with regard to revegetation and combatting desertification (Fig. 6).

### Importance of maximum CV magnitude for understanding vegetation change

The month of maximum CV magnitude represents the key period for vegetation change, which can serve as a decision-making guide for combatting vegetation degeneration or desertification. Two general regions can be identified in which the months of maximum CV magnitude show different behaviors. One is the region of gradual change in the middle and western part of the study area; the other is in the western part of the study are, called the chaos region.

The regions from the Taklimakan Desert to the Qaidam Basin and from the Gurban Tonggut Desert to the Tenger Sandy land belong to the chaos region and are characterized by desert, in which vegetation cover depends on unreliable rainfall. The region of gradual change is characterized by distinct months of maximum CV magnitude. Seven areas within this region were selected to better understand key periods for vegetation change during the growing season. In the regions from Mt. Yinshan to Mt. Luliang, Mt. Taihang, and the Haihe River basin, the months of maximum CV magnitude are July (Mt. Yinshan), June (western Mt. Yinshan), May (northern Haihe River Basin and eastern Mt. Taihang), September (western Haihe River Basin), and July (eastern Haihe River basin). In the region from the Loess Plateau to the Guanzhong Basin, the months of maximum CV magnitude are August (northern Loess Plateau), June (middle to southern part of the Loess Plateau), May (southern Loess Plateau), September (central Guanzhong Basin), and April (areas surrounding Guanzhong Basin). In the region from Mt. Xiaohingganling and Mt. Changbai to the Northeast Plain and Horqin Sandy Land, the months of maximum CV magnitude are May (Mt. Xiaohingganling and Mt. Changbai), September (area south of Mt. Xiaohingganling, Northeast Plain and Three River Plain), July (area south of Mt. Xiaohingganling and west of Northeast Plain), and August (area surrounding Horqin Sandy Land). In the region of Mt. Dahingganling, maximum CV magnitudes occur, from north to south, in October, April, June, and August. In the region from Mt. Dahingganling, along the Hulunbuir Plateau to Mt. Henteyn, the months of maximum CV magnitude are June (west of Mt. Dahingganling), July (east of the Hulunbuir Plateau), July–August (central Hulunbuir Plateau), August to September (west of the Hulunbuir Plateau), June–July (area east of Mt. Henteyn), and October (central Mt. Henteyn). In the regions from Mt. Hentyn along the Desert Basin to the Mongolian Plateau, the months of maximum CV magnitude are October (Mt. Henteyn), July to September (area south of Mt. Henteyn), September (area north of Desert Basin), August (between the Desert Basin and Mongolian Plateau), and August to September (area east of the Desert Basin, the Mongolian Plateau, and Mt. Gobi Altayn). In the regions from northern Mongolia along Mt. Hangayn to Mt. Altayn, the months of maximum CV magnitude are October to September (northern Mongolia), July to August (between Mt. Altayn and Mt. Hangayn), and August (central Mt. Altayn).

## Conclusion

We have used *CV analysis* to determine months of maximum CV magnitudes in an attempt to better understand vegetation change through remote sensing and high-resolution NDVI time series data. The CV magnitude can serve as an indicator for detecting vegetation change, which can be used for future event prediction. Eleven regions in northern China and Mongolia were investigated and typical vegetation change periods were shown for each region. The months of maximum CV magnitude help us understanding vegetation growing conditions in different years, which will enhance our ability to prevent desertification and promote revegetation. Our study area is an arid and semi-arid region, where taking the analysis between annual precipitation/solar radiation and vegetation dynamic seems not have real meaning although there might have some kinds of R or P values. In the area, vegetation could grow very rapid once the rainfall occurred, after the rainfall, the vegetation would go very quickly once the water consumed. We thus not taking the correlation analysis, but we will consider the ways of how to connect annual precipitation/solar radiation and vegetation dynamic in the future when we recover the real relationship between them by field observations.
